# Could positive airway pressure enhance brain waste clearance and modify neurodegenerative risk? A perspective on sleep-dependent cerebrospinal fluid–lymphatic pathways

**DOI:** 10.1093/sleep/zsag065

**Published:** 2026-03-06

**Authors:** Heon-Jeong Lee

**Affiliations:** Department of Psychiatry, Korea University College of Medicine, Seoul, Republic of Korea; Chronobiology Institute, Korea University, Seoul, Republic of Korea

**Keywords:** obstructive sleep apnea, positive airway pressure, cerebrospinal fluid, glymphatic system, meningeal lymphatics

## Abstract

Obstructive sleep apnea is linked to cognitive decline and is increasingly implicated in neurodegenerative trajectories. Continuous positive airway pressure, the most common form of positive airway pressure used to treat obstructive sleep apnea, improves breathing and sleep continuity, yet cognitive outcomes, as well as reports of rapid eye movement sleep behavior disorder manifestations in comorbid obstructive sleep apnea, remain heterogeneous. In parallel, the last decade has reframed sleep as an active state for brain fluid transport, in which cerebrospinal fluid movement, vascular and respiratory mechanics, and extracranial lymphatic drainage jointly influence the clearance of potentially neurotoxic solutes (e.g. amyloid-β). This Perspective advances a testable, conditional hypothesis: beyond correcting hypoxemia and sleep fragmentation, positive airway pressure (and specifically continuous positive airway pressure) may modulate cerebrospinal fluid–lymphatic clearance by changing upper-airway pressure gradients, intrathoracic pressure swings, craniovenous pulsatility, and extracranial lymphatic outflow mechanics. This Perspective also reviews counterevidence, including human physiologic data showing reduced cerebrospinal fluid stroke volume under higher-pressure continuous positive airway pressure during wakefulness and persistent heterogeneity in cognitive outcomes. This Perspective proposes a mechanistic framework that separates (1) event suppression (restored consolidated sleep), (2) pressure-dependent cerebrospinal fluid/venous coupling, and (3) route-dependent extracranial lymphatic outflow as partially independent modules. Finally, this Perspective outlines a focused research agenda integrating polysomnography, fast neuroimaging, perivascular transport metrics (e.g. diffusion tensor imaging-analysis along the perivascular space), and cerebrospinal fluid/blood biomarkers to determine when, for whom, and through which pathways positive airway pressure could exert neuroprotective effects.

## Graphical Abstract

**Graphical Abstract:**
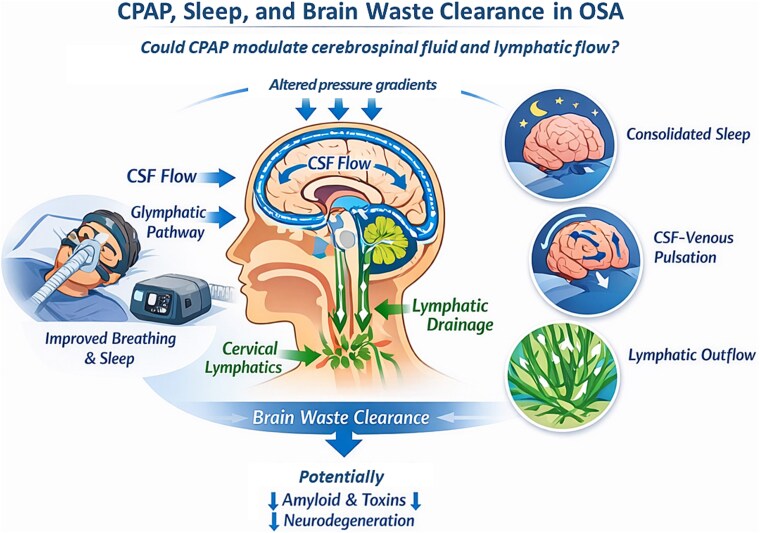

## Introduction

Obstructive sleep apnea (OSA) is among the most prevalent sleep disorders and is characterized by repetitive upper-airway collapse, sleep fragmentation, and intermittent hypoxemia. Beyond daytime sleepiness and cardiometabolic morbidity, OSA is increasingly framed as a brain-health disorder: epidemiologic studies associate OSA with poorer cognitive performance and increased risk of mild cognitive impairment and dementia, although effect sizes vary across cohorts and analytic approaches [1, 2].

Mechanistic accounts typically emphasize intermittent hypoxia, intermittent hypercapnia, sympathetic surges, oxidative stress, vascular injury, and, critically, sleep fragmentation and sleep-architecture disruption. A complementary (and potentially modifiable) pathway is sleep-dependent brain fluid transport and “waste clearance.” In animal models, sleep accelerates the removal of interstitial solutes, including amyloid-β, through cerebrospinal fluid (CSF)–interstitial exchange along perivascular routes (the glymphatic framework) [3, 4]. Human work has identified large, low-frequency CSF oscillations during non-rapid eye movement (REM) sleep and strong coupling between respiration and CSF flow [5, 6].

Positive airway pressure (PAP) (most commonly continuous positive airway pressure [CPAP]) is first-line therapy for OSA, but its effects on neurocognition are heterogeneous across trials and cohorts [7, 8], and in patients with comorbid OSA and rapid eye movement sleep behavior disorder (RBD), CPAP has been reported to reduce dream-enactment behaviors in some studies [9, 10]. Two recent lines of human evidence sharpen the clinical relevance of sleep-dependent clearance biology for OSA. In an Alzheimer’s Disease Neuroimaging Initiative (ADNI)-based longitudinal analysis, self-reported OSA was associated with faster biomarker change in cognitively normal and mildly impaired adults, including greater annual amyloid Positron Emission Tomography (PET) accumulation and worsening CSF amyloid/tau trajectories over follow-up [11]. Separately, magnetic resonance imaging (MRI) studies suggest impaired putative glymphatic function in OSA, including reduced diffusion tensor imaging-analysis along the perivascular space (DTI-ALPS) compared with controls [12]; a recent meta-analysis of severe OSA synthesizes the DTI-ALPS literature and highlighted key sources of heterogeneity and bias in current evidence [13]. Together, these findings motivate a provocative question for sleep medicine: can treating OSA with CPAP do more than normalize breathing; could it also shift CSF–lymphatic clearance and thereby potentially influence long-term neurodegenerative risk?

### Terminology

This Perspective uses PAP as an umbrella term for therapies delivering pressurized air to the upper airway (CPAP, auto-adjusting PAP, bilevel PAP). This Perspective refers to CPAP specifically when evidence pertains to the continuous mode or fixed-pressure protocols.

### Core perspective

**Figure 1:**
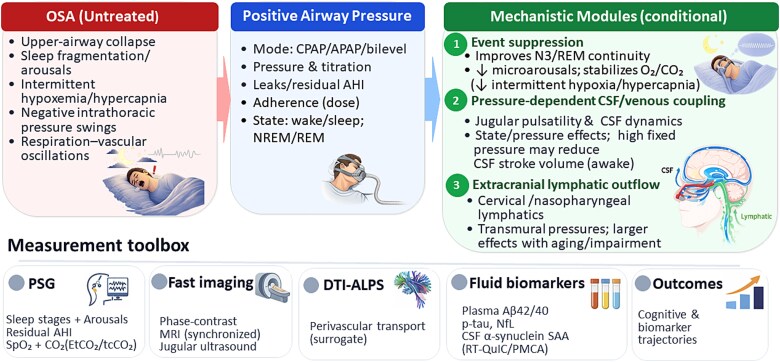
A modular framework linking positive airway pressure to sleep-dependent CSF–lymphatic clearance. Conceptual schematic summarizing how positive airway pressure (PAP) may influence sleep-dependent cerebrospinal fluid (CSF)–lymphatic pathways through three partially independent modules: (1) event suppression (reduced respiratory events, arousals, and blood-gas instability including intermittent hypoxia/hypercapnia), (2) pressure-dependent CSF/venous coupling (altered intrathoracic pressure, venous return, and CSF pulsatility), and (3) route-dependent outflow (shift in extracranial lymphatic drainage). Potential downstream implications for neurodegenerative risk are hypothesized to depend on state dependence, pressure profile, and outflow route. Measures shown are illustrative; DTI-ALPS is an indirect surrogate of perivascular water movement rather than a direct clearance measure, and fluid biomarkers can be assessed in plasma/serum with optional CSF substudies depending on cohort feasibility. Abbreviations: APAP, auto-adjusting positive airway pressure; N3, stage N3 (slow-wave sleep); NfL, neurofilament light; PMCA, protein misfolding cyclic amplification; PSG, polysomnography; p-tau, phosphorylated tau; RT-QuIC, real-time quaking-induced conversion; SAA, seed amplification assay; SpO₂, peripheral capillary oxygen saturation.

PAP’s net effect on sleep-dependent brain waste clearance is unlikely to be uniformly beneficial or harmful. The dominant benefit is probably indirect, via the suppression of obstructive events that restores slow-wave/REM continuity and stabilizes respiratory–vascular oscillations; yet positive pressure may also directly change venous and CSF dynamics in a state- and pressure-dependent manner. This Perspective outlines a modular, testable framework to integrate these effects and to guide physiologic and biomarker studies. Figure 1 summarizes the proposed modular model, linking untreated OSA features, PAP treatment characteristics, three conditional mechanistic modules, and a measurement toolbox to test clearance-related outcomes.

This translational question may be especially relevant for synucleinopathies. Idiopathic REM sleep behavior disorder (iRBD) is a prototypical prodromal α-synucleinopathy, and OSA frequently co-occurs. Small observational cohorts and a recent systematic review suggest that treating comorbid OSA with CPAP may reduce the frequency or severity of dream enactment behaviors in some patients with concurrent RBD and OSA, although outcomes are heterogeneous and often based on self-reported measures [9, 10]. In Parkinson disease, observational studies report that patients with OSA show progression of cognitive and motor decline, whereas those treated with CPAP may show a more favorable trajectory [14, 15]; a randomized controlled trial also suggests improved cognitive outcomes with PAP in this group [16]. In parallel, a large electronic health record-based cohort study in US veterans reported that OSA was associated with incident Parkinson disease and that this association was weaker among individuals with evidence of PAP treatment; these findings are observational and may be influenced by residual confounding, but they motivate prospective studies that test whether PAP-related physiological changes track with α-syn–linked biomarkers or phenoconversion in prodromal populations [17]. Preclinical work further suggests that impaired glymphatic or meningeal lymphatic clearance may contribute to α-syn accumulation and spread, providing a mechanistic rationale for examining sleep-dependent clearance pathways in synucleinopathies [18].

## Sleep-Dependent CSF Movement and Extracranial Outflow

Sleep-dependent clearance is not a single pathway but a family of coupled processes spanning fluid exchange, solute transport, and outflow pathways. In the glymphatic framework, CSF enters along periarterial routes, exchanges with interstitial fluid via astroglial water channels, and exits along perivenous pathways carrying solutes such as amyloid-β [3, 4].

In humans, dynamic CSF signals have provided a complementary lens to tracer-based clearance. Non-rapid eye movement sleep is accompanied by prominent CSF oscillations temporally aligned with slow hemodynamic and electrophysiologic fluctuations, consistent with a coordinated pumping milieu [5]. Respiration is another major driver: inspiration-related pressure changes can promote craniospinal CSF shifts [6].

Where does CSF go after it moves? Meningeal lymphatic vessels provide a structural and functional route for CNS lymphatic drainage [19, 20], and aging appears to impair meningeal lymphatic function with relevance to AD models [21]. More recently, mapping studies have strengthened the case for extracranial routes, including the deep cervical lymphatics and a nasopharyngeal lymphatic plexus, as important candidate CSF outflow hubs [22], raising the possibility that peripharyngeal mechanics and neck structure could, in theory, be influenced by airway pressure changes. Non-invasive mechanical activation of superficial cervical lymphatics can also increase CSF drainage in aged animals [23].

## A Conditional Hypothesis: How PAP Could Help or Hinder Clearance

### Pro case (event suppression and stabilization of sleep/respiration)

OSA destabilizes sleep-stage continuity and produces large intrathoracic pressure swings that can perturb venous return and craniospinal adherence (the system’s ability to accommodate intracranial volume changes, such as those from vascular or CSF shifts, while maintaining stable pressure). By suppressing obstructive events, CPAP typically consolidates sleep, reduces arousal impact (sleep fragmentation), improves oxygenation, and reduces extreme negative intrathoracic pressure swings. Because blood-gas fluctuations (including intermittent hypercapnia) can influence vasomotor tone and glymphatic–lymphatic coupling, future mechanistic studies should measure and control for CO₂/O₂ dynamics alongside sleep stage and PAP settings [24]. In anesthetized rodents, CPAP increased CSF flow speed and augmented regional glymphatic transport, supporting biological plausibility for a clearance-enhancing effect under some conditions [25].

### Con case (pressure-dependent mechanical coupling and uncertain routes)

In healthy awake volunteers studied with phase-contrast MRI at the craniovertebral junction, CPAP at 15 cmH₂O reduced spinal CSF stroke volume and pulsation amplitude and altered jugular venous flow, consistent with pressure-dependent craniospinal–venous coupling that could oppose some components of CSF dynamics under certain settings, at least in wakefulness and at higher pressures [26]. Separately, clinical studies of CPAP and cognition show mixed results and are strongly influenced by adherence, exposure duration, sleep restoration, and baseline neurodegenerative risk [7, 8]. Accordingly, the premise that CPAP enhances clearance should be treated as a conditional hypothesis with explicit mediators and boundary conditions, including pressure level, sleep stage, and concurrent blood-gas dynamics [24].

## A Modular Mechanistic Framework to Explain the Apparent Conflict

This Perspective proposes a pragmatic framework in which PAP influences clearance through partially separable modules, each with distinct state dependence and mediators.

Module 1: Event suppression (likely dominant). By eliminating obstructive events, PAP restores consolidated sleep and improves oxygenation. If this module dominates, clearance-related endpoints should track with improvements in sleep continuity, reduced microarousals, and lower residual apnea-hypopnea index (AHI), largely independent of the exact pressure profile.

Module 2: Pressure-dependent CSF/venous coupling. PAP changes upper-airway and intrathoracic pressures, potentially altering venous return, jugular venous pulsatility, craniovenous adherence, and the balance between respiratory- versus vascular-driven CSF motion. If this module dominates, different PAP modes and titration levels could produce different CSF dynamic patterns even when AHI is similarly controlled. This module is illustrated by awake volunteer data showing that high fixed-pressure CPAP can reduce spinal CSF stroke volume and jugular venous pulsation amplitude [26].

Module 3: Route- and structure-dependent extracranial lymphatic outflow. If nasopharyngeal and cervical lymphatic pathways are important CSF outflow routes, PAP may influence lymphatic resistance or pumping by changing transmural pressures in peripharyngeal tissues. Conceptually, raising tissue (external) pressure around compliant lymphatic collectors would reduce lymphatic transmural pressure and could narrow the lumen, increasing resistance and potentially impairing intrinsic pumping; conversely, if PAP raises upstream CSF/venous pressures and increases lymphatic luminal pressure, transmural pressure could increase and favor patency or flow. Because these directions cannot be inferred a priori in humans, studies should directly quantify lymphatic caliber/flow (when feasible) and relate it to PAP pressure level, neck structure, and sleep stage. Effects may be larger in older adults or in those with impaired lymphatic function, and could be studied alongside interventions that mechanically activate superficial cervical lymphatics (e.g. as shown in recent rodent models [23]).

## A Focused Research Agenda for SLEEP

1. Measure CSF and venous dynamics during natural sleep (not only wakefulness) with within-subject comparisons of untreated OSA nights versus titrated PAP nights. Combine polysomnography with fast imaging (e.g. phase-contrast MRI synchronized to respiratory/cardiac signals) or ultrasound measures of jugular flow across sleep stages.
2. Integrate dynamic CSF/venous measures with imaging surrogates of perivascular transport. DTI-ALPS and related MRI metrics are indirect surrogates of perivascular water movement and can be sensitive to acquisition and processing; they should be interpreted as markers of putative glymphatic function rather than direct “clearance” measures. Studies should test whether these metrics change after sustained PAP adherence and whether changes correlate with sleep-stage restoration [12].
3. Track molecular biomarker trajectories longitudinally. A tiered approach could follow plasma amyloid-β 42/40 ratio, phosphorylated tau, and neurofilament light in large PAP-treated and untreated cohorts, with CSF biomarker substudy sampling in mechanistic subsets. Biomarker analyses should be enriched for high-risk individuals (older age, Apolipoprotein E (APOE) ε4, high baseline amyloid/tau impact) [11]. In synucleinopathy-enriched cohorts (e.g. iRBD with comorbid OSA), consider incorporating α-synuclein–relevant biomarkers (eg, CSF or peripheral α-syn seeding assays) and time-to-phenoconversion endpoints alongside Alzheimer-type biomarkers to explore whether PAP-related physiological changes covary with α-syn–linked biological or clinical trajectories [17, 18].
4. Phenotype the intervention. PAP mode, pressure settings, leaks, residual AHI, and objectively measured adherence should be reported consistently, because mechanistic effects are likely pressure- and state-dependent and may differ at higher fixed pressures that measurably alter CSF/venous pulsatility in awake volunteers [8, 26–28].

### Clinical implications

While clinicians should continue to treat OSA with PAP for established benefits, future trials should evaluate potential neuroprotective mechanisms using sleep-stage–resolved physiologic endpoints (CSF and jugular venous dynamics), candidate imaging surrogates (e.g. DTI-ALPS), and longitudinal fluid biomarkers. The most informative comparisons will stratify by pressure profile, achieved sleep restoration, outflow pathway structure, and objectively measured adherence, not simply PAP versus no PAP.

## Conclusion

PAP is an established, effective treatment for OSA. The open question is not whether PAP works, but whether and when it can also influence sleep-dependent CSF–lymphatic clearance in ways relevant to long-term brain health. A conditional, modular view helps explain apparently conflicting physiologic and clinical data and leads to testable predictions with measurable mediators. Testing these predictions will require integrative studies that align polysomnography with fluid-dynamic imaging, perivascular transport metrics, and molecular biomarkers, moving the field from plausible narratives to robust mechanistic evidence.

## Disclosure statement

*Financial disclosure*: This work was supported by the Bio&Medical Technology Development Program of the National Research Foundation of Korea (MSIT) (No. RS-2024-00440371).

*Non-financial disclosure:* H.-J.L. is a cofounder and CTO of hucircadian; this work has no financial or non-financial relationship with hucircadian, and hucircadian had no role in the conception, design, analysis, interpretation, or writing of this manuscript.

## AI tool use

Portions of language editing and formatting were assisted by an AI language model. Figure 1 was prepared with the assistance of an AI-based image generation tool; the author reviewed, verified, and edited the figure. The author takes full responsibility for the content and figures.
